# Motor Vehicle Accident Eye Injuries in Northern Israel

**DOI:** 10.3390/ijerph110404311

**Published:** 2014-04-17

**Authors:** Michael Yulish, Joseph Pikkel

**Affiliations:** 1 Ziv Medical Center, Safed, 13110, Israel; 2 Faculty of Medicine, Bar Ilan University, Safed, 13150, Israel; E-Mail: pikel.y@ziv.health.gov.il

**Keywords:** motor vehicle accident, eye injury, seatbelt, air bag

## Abstract

*Purpose*: To investigate the occurrence and types of motor vehicle accident eye trauma in north Israel. *Methods*: The records between the years 2007–2011 of the Ophthalmology Emergency Room of one medical center were searched. Eye injuries due to motor vehicle accidents were classified according to type, severity of injury and demographic data of patients. *Results*: Nearly five percents of ER presentations were due to motor vehicle accidents. Most motor vehicle accident-related eye injuries were mild. *Conclusion*: Efforts should be taken to prevention and to minimize the severity of motor vehicle accident-related eye injuries.

## 1. Introduction

Of all causes of visual impairment, eye injury is among the most preventable. Motor vehicle accidents are responsible for a considerable number of eye injuries [1]. Seat belt use was found to be associated with a 2-fold reduction in eye injury risk [1]. Penetrating eye injuries from road traffic accidents were shown to decrease considerably after seatbelt legislation and the introduction of laminated windscreens [2,3]. Though frontal air bag deployment was found to be associated with a 2-fold increased risk of eye injury [1], its protection against serious injury justifies its use; fewer severe injuries were found to occur among occupants exposed to air bag deployment [4]. Analysis according to type of injury showed a statistically significant increased risk of corneal abrasions for occupants exposed to air bag deployment (*p* = 0.03) [4]. Air bag deployment was found to be associated with traumatic maculopathy [5]. Nevertheless, the air bag seems to have decreased the risk of severe eye injuries. A one year surveillance study in Malaysia reported that motor vehicle accidents accounted for 23.1% of the incidents of eye trauma [6]. The severe eye penetrating injury is associated with non use of seatbelts [1,2,3]. Panaqiotidis *et al.* [7] showed only 5.2% of the persons suffering from severe eye injury used safety belts. On the other hand, only a small proportion of motor vehicle injuries involve the eye (18 per 1,000 occupants) [1]. Nevertheless, given the number of motor vehicle accidents that occur each year, the number of related eye injuries is large. Considering all eye injuries, skin and soft tissue injuries (*i.e.*, eyelids) are most common (88.4%), corneal abrasions and conjunctival injuries account for 5.7% and 3.3%, respectively; optic nerve injury, corneal laceration, scleral rupture and tear duct laceration are less frequent (0.09%, 0.4%, 0.21%, 0.05%) [1]. Regarding motor vehicle accidents, occupants with higher body weight have been reported to have a reduced risk of eye injury [1]. The risk of eye injury is highest for frontal collisions, with the most common injury mechanism the windshield, followed by the frontal air bag, steering wheel, and flying glass [1]. An increasing proportion of the population undergoes laser refractive surgery. Studies indicate that such surgical procedures weaken the cornea and make it more susceptible to injury for years after the procedure [8,9], especially, to injury associated with air bag deployment. The current study analyzed and classified MVA-related eye injuries in northern Israel.

## 2. Methods

The facilities of Ziv Medical Center, Safed, Israel serve the population of the Galilee, Golan Heights and Sea of Galilee, nearly one hundred and eighty thousand people. The medical records of the Ophthalmology Emergency Room between 1 January 2007 and 31 December 2011 were searched. Eye injuries due to motor vehicle accidents were classified according to type, severity of injury and demographic data of patients. Motor vehicle accident-related eye injuries were analyzed and classified according to severity of injury and demographic data.

## 3. Results

A total of 6,783 motor vehicle accident occupants were evaluated in the general Emergency Room of Ziv Medical Center, Safed, Israel during 2007–2011. Of them, 865 (12.75%) were evaluated in the Ophthalmology Emergency Room. The number of patients evaluated in the Ophthalmology Emergency Room during the study period, and the proportion who suffered from motor vehicle accident-related eye injuries is presented on Table 1. Every year more than three thousand patients were examined in the Ophthalmology Emergency Room. About 5% of the injuries were due to motor vehicle accidents. Demographic data of those with eye injuries are summarized in Table 2. Nearly two-thirds were male, similarly to Panaqiotidis *et al.* results [9]. There was a tendency toward younger age, both for men and women. Of all patients examined due to motor vehicle accident-related eye injury, 128 (14.8%) were hospitalized. The types of injuries are summarized in Table 3. Most were mild, including subconjunctival hemorrhage, corneal erosions, lid lacerations and hyphema (Table 3). For 21% of patients the eye examination did not reveal a pathological finding. Few suffered from severe injuries, including orbital fractures (0.9%), perforating injuries (1%) and optic nerve or choroidal rupture (0.3%). Sixty-nine patients (8%) were evaluated due to air bag injuries. Air bag injuries were mostly mild, including 47 (5.4%) corneal erosions, 33 (3.8%) hyphema, and 24 (2.8%) subconjunctival bleeding. However, four orbital fractures were caused due to air bag injuries (Table 4).

**Table 1:** Ophthalmology Emergency Room statistics during 2007–2011.

Year	2007	2008	2009	2010	2011	Overall
Ophthalmology ER (number of patients)	3,123	3,274	3,450	3,573	3,571	16,991
Patients injured due to motor vehicle accidents (number/%)	158/5.1%	186/5.7%	162/4.7%	178/5.0%	181/5.1%	865/5.1%

**Table 2:** Demographic data of MVA-related eye injuries in 2007–2011.

Gender	Number and Age Distribution
Male (Number**/%)**	561/64.8%
Female (Number/%)	304/35.1%
Male: Mean age (year) ± SD, Age Range (year)	29 ± 11.2, 8–67
Female: Mean age (year) ± SD, Age Range (year)	20 ± 4.0, 12–58

**Table 3:** Types of eye injuries.

Type of Eye Injury	Number	%
Subconjunctival hemorrhage	294	34
Corneal erosion	234	27
Lid laceration	156	18
Orbital fractures	8	0.9
Hyphema	176	20.3
Perforating injuries	9	1
Diplopia	4	0.5
Optic nerve or choroidal rupture	3	0.3
No finding	182	21

**Table 4:** The Distribution of Airbag eye injuries according to the severity of injury.

Type of the Injury	Subconjunctival Hemorrhage	Hyphema	Corneal Erosion	Orbital Fractures
Number	24	33	47	4
%	2.8	3.8	5.4	0.5

## 4. Discussion

The current study shows occurrence and type of motor vehicle-related eye injuries in northern Israel in 2007–2011. Each year during the five year study period, more than three thousand patients were examined in the Ophthalmology Emergency Room of Ziv Medical Center, Safed, Israel; 5% of them due to motor vehicle accidents. Eye injuries were diagnosed in 79% (683/865) of motor vehicle accident occupants who were evaluated in the Ophthalmology Emergency Room during 2007–2011, which is 10% (683/6,783) of the occupants who arrived to the hospital following a motor vehicle accident. This is considerably higher than the 1.8% rate reported in the 1988–2001 United States National Automotive Sampling System Crashworthiness Data System files [1]. We do not have an explanation for the particularly high rate of eye injury observed in the current study. Perhaps lower compliance with seat belt laws may provide a partial explanation. Most MVA-related eye injuries in the current study, as well as in the U.S. survey, were mild [1]. Nevertheless, there were severe injuries, such as optic nerve and choroidal rupture, perforating injuries and orbital fractures. In this study, most eye injuries due to airbag deployment were mild, yet four orbital fractures were caused by airbag deployment. 

## 5. Conclusions

About 5% of patients referred to the ophthalmic ER in the Ziv Medical Center are due to motor vehicle accidents. While most motor vehicle accident-related eye injuries were mild, the rate of injuries was high. Seatbelt and airbag usage is important for preventing and reducing the severity of motor vehicle related eye injuries.
